# Agreement Between Caregivers' Concerns of Children's Developmental Problems and Professional Identification in Taiwan

**DOI:** 10.3389/fped.2022.804427

**Published:** 2022-02-28

**Authors:** Ling-Yi Lin, Wen-Hao Yu, Wei-Pin Lin, Chih-Chia Chen, Yi-Fang Tu

**Affiliations:** ^1^Department of Occupational Therapy, College of Medicine, National Cheng Kung University, Tainan City, Taiwan; ^2^Institute of Allied Health Sciences, College of Medicine, National Cheng Kung University, Tainan City, Taiwan; ^3^Department of Pediatrics, National Cheng Kung University Hospital, College of Medicine, National Cheng Kung University, Tainan City, Taiwan; ^4^Institute of Clinical Medicine, College of Medicine, National Cheng Kung University, Tainan City, Taiwan; ^5^Department of Physical Medicine and Rehabilitation, National Cheng Kung University Hospital, College of Medicine, National Cheng Kung University, Tainan City, Taiwan

**Keywords:** developmental delay, caregiver's concerns, professional identification, agreement, speech delay and global developmental delay

## Abstract

**Objective:**

Early detection of developmental delays relies on the accuracy of the caregivers' concerns of children's developmental problems. The aim of this study was to investigate the agreement between the caregivers' awareness of children's developmental problems and professional identification.

**Methods:**

Caregivers of 1,963 children (age range: 5–71 months; mean: 38.4 months) younger than 6 years old who were at risk of developmental delays and referred to the center for a comprehensive evaluation were enrolled in this study. Children were identified by a transdisciplinary team including a pediatric neurologist, a pediatric psychiatrist, two psychologists, two occupational therapists, two physical therapists, two speech therapists, a social worker, and a special instructor. A series of standardized developmental assessments were used to identify children with developmental delay. Retrospective chart reviews were conducted on all children to confirm specific developmental disorders.

**Results:**

The caregivers' initial concerns of cognitive, speech/language, emotional/behavioral, and motor and global development showed low agreement with the results of professional identification. The major disagreement was observed in the cognitive domain. Speech/language developmental concern was an important red-flag indicator of cognitive and emotional/behavioral developmental delays. The presence of intellectual disability, autism spectrum disorder, and attention deficit hyperactivity disorder was high in this study. When having caregivers' concerns about speech/language and emotional/behavioral development, the odds of children with autism spectrum disorder was 2.37 and 2.17 times greater than those without autism spectrum disorder, respectively. The presence of attention deficit hyperactivity disorder was significantly associated with concerns about cognitive and emotional/behavioral developmental delays. Child's age and mothers' level of education were significant indicators for detecting the child's developmental problems.

**Conclusion:**

It is recommended that assessing the cognitive developmental status is essential for all children in the identification process. Practitioners should not overlook caregivers' concern about speech/language and emotional/behavioral development. Transdisciplinary practitioners provide educational guidance to caregivers, especially in the domains of cognitive, speech/language, and emotional/behavioral development.

## Introduction

Early detection of developmental delays allows the timely provision of appropriate early intervention services (1, 2). Previous research has indicated that parental concerns using a screening approach for developmental delay offer substantial data in early childhood (3–5); however, often many parents do not have the sufficient opportunity to share their child's developmental concerns with their pediatrician (1, 4, 6).

Caregiver awareness is one of the various factors that determine the prognosis or eventual outcome of children with developmental delay (7). While the accuracy of parental awareness of children's developmental status remains controversial, it is generally accepted that parents have a key role to play in the identification process (8, 9). Some studies have shown a high level of agreement between caregiver awareness and clinical diagnosis (10, 11). Caregivers' concerns about language, emotional/behavioral, and motor developmental problems are valid indicators of children's developmental status, but identifying cognitive or global developmental problems correctly might be difficult for some caregivers (10, 12).

For over a decade, a significant decline in the prevalence of developmental delays was observed in US children aged 3–17 years from 2009 to 2011 and 2015 to 2017 (4.65–4.06%; a decline of 12.7%) (13). The associations between caregiver awareness in multiple developmental problems and professional assessment in cognitive developmental delay have been established in Taiwan (14). A study reported the agreement between caregiver concerns and professional assessments, reporting high agreement between caregivers and professionals in the global and the motor domains and low agreement in the cognitive, speech/language, and emotional/behavioral domains (15).

Co-occurrence of specific developmental disorders has been recognized in children with global developmental delay (10). The most common developmental disorders include intellectual disability (ID), cerebral palsy, autism spectrum disorder (ASD), attention deficit hyperactivity disorder (ADHD), hearing loss, and learning disability, with significant increases in the prevalence of ADHD (8.5–9.5%, *p* < 0.01), ASD (1.1–2.5%, *p* < 0.001), and ID (0.9–1.2%, *p* < 0.05) from 2009 to 2017 (13). Young children with ADHD had clinically significant co-occurring delays in multiple developmental domains (16, 17). The prevalence of ASD is about 1 in 54, but this is expected to increase (18) and has increased dramatically over the past 25 years in Taiwan (19, 20). More researchers have paid attention to parental awareness of ASD since many parents experienced an arduous, lengthy, and fraught process of obtaining an ASD diagnosis (21–23). Some studies have also investigated the relationships between parental concerns and ASD (10, 24, 25), with the co-occurrence of ASD significantly associated with parental concerns about language, behavioral, or global developmental problems in Taiwan (10). However, their conclusions were drawn based on modest-sized samples.

Several demographic characteristics such as maternal education have been associated with identifying developmental delays (25, 26), with the mothers' educational levels affecting the accuracy of the detection of developmental delays in their children (15, 27), whereas others observed no such relationship (28). Parents with higher educational levels may be more likely to express their developmental concerns verbally than those with lower educational levels (29). There were inconclusive results regarding the relationship between mothers' education levels and detection of developmental delays and may need further investigation.

The purposes of this study were to address three research questions: (1) Do caregivers' concerns of children's developmental problems agree with the professional identification? (2) To what extent do the relationships between caregivers' concerns and specific developmental disorders exist? (3) Do demographic characteristics account for the variability in caregivers' concerns of children's developmental problems? Having a better understanding of the agreement between caregivers' concerns of children's developmental problems and the results of professional identification will help pediatric practitioners provide parenting education and early intervention services.

## Methods

### Design

The design of the study was a retrospective cohort study.

### Participants

Data were drawn from 1,963 children at risk for/with developmental delay and their caregivers through the Center of Team Evaluation for Children's Development in southern Taiwan from January 2017 to December 2019. Children under 6 years old at risk of developmental delays who were referred to the center for a comprehensive evaluation were enrolled in the study. Many parents had concerns about their child's condition due to developmental disorders and health. They would ask pediatricians for a referral to the center for a comprehensive evaluation. Some children were referred by preschool teachers when they failed screening. Certain children were referred by pediatricians while at the clinic visit. Initially, the case manager interviewed caregivers and obtained written informed consent. The case manager used a standard set of checklist items to categorize caregiver concerns. Next, children were evaluated by a transdisciplinary team including a pediatric neurologist, a pediatric psychiatrist, two psychologists, two occupational therapists, two physical therapists, two speech therapists, a social worker, and a special instructor. After the evaluation by the transdisciplinary team, the pediatric neurologist and pediatric psychiatrist would give the diagnosis to children based on the International Classification of Diseases, 10th revision (ICD-10) codes and documented on the medical chart. Table 1 presents the demographic and family characteristics of the participants. A single domain concern refers to only one developmental problem reported by a caregiver. A multiple domain concern refers to at least two developmental problems reported by a caregiver. There were 113 (5.8%) married immigrant families. Families of migrants were from mainland China (*n* = 57), Vietnam (*n* = 33), Philippines (*n* = 5), Indonesia (*n* = 4), Korea (*n* = 3), Thailand (*n* = 2), Malaysia (*n* = 1), India (*n* = 1), Japan (*n* = 1), Egypt (*n* = 1), Croatia (*n* = 1), Australia (*n* = 1), Sweden (*n* = 1), Netherlands (*n* = 1), and United States (*n* = 1).

**Table 1:** Sample and family characteristics (*N* = 1,963).

**Characteristics**	**Single domain concern** **(***n*** = 1,086)**	**Multiple domain concern** **(***n*** = 877)**	**Total**
	**Mean (SD)/***n*** (%)**	**Mean (SD)/***n*** (%)**	**Mean (SD)/***n*** (%)**
Age (months)	34.6 (14.9)	43.1 (14.7)	38.4 (15.4)
5–35 months	680 (62.6%)	278 (31.7%)	958 (48.8%)
36–71 months	406 (37.4%)	599 (68.3%)	1,005 (51.2%)
**Gender**			
Male	773 (71.2%)	664 (75.7%)	1,437 (73.2%)
Female	313 (28.8%)	213 (24.3%)	526 (26.8%)
Preterm	157 (14.5%)	141 (16.1%)	298 (15.2%)
First child in the family	666 (61.3%)	570 (65.0%)	1,236 (63.0%)
Number of children in the family	1.7 (0.7)	1.7 (0.7)	1.7 (0.7)
**Father**			
Age (years)	37.3 (5.5)	37.7 (5.7)	37.5 (5.6)
**Level of education**			
Bachelor degree or above	384 (35.4%)	355 (40.5%)	1,224 (62.4%)
High school and below	702 (64.6%)	522 (59.5%)	739 (37.6%)
**Mother**			
Age (years)	34.6 (4.9)	34.9 (5.0)	34.8 (4.9)
**Level of education**			
Bachelor degree or above	353 (32.5%)	328 (37.4%)	1,282 (65.3%)
High school and below	733 (67.5%)	549 (62.6%)	681 (34.7%)
**Marriage**			
Taiwanese couples	1,026 (94.5%)	824 (94.0%)	1,850 (94.2%)
Immigrant couples	60 (5.5%)	53 (6.0%)	113 (5.8%)

### Measures

Caregivers were interviewed by a case manager about their concerns and the demographic characteristics of the children and family. When interviewing, one parent was present and mainly was the mother of the child. The caregivers' concerns were categorized into five developmental domains: cognition, speech/language, emotion/behavior, motor, and global. Global developmental problems were coded when caregivers reported at least two or more developmental domains of problems. A series of developmental assessments including the Bayley Scales of Infant Development-Third edition (Bayley-III), Wechsler Preschool and Primary Scale of Intelligence-Revised (WPPSI-R), Peabody Developmental Motor Scale-Second edition (PDMS-2), Chinese Children Developmental Inventory (CCDI), the Screening Tool for Autism in Toddlers and Young Children (STAT), and Child Behavior Checklist (CBCL) were used to identify children with developmental delay. The registered psychologists used the Bayley-III, WPPSI-R, and STAT. Occupational therapists and physical therapists used the PDMS-2. Main caregivers filled out the CCDI and CBCL. All of the tests listed were done on all the included children in a standardized manner. Retrospective chart reviews were conducted on all children to confirm a diagnosis on ID, ASD, and ADHD based on the ICD-10 codes.

### Data Analysis

The demographic data, independent variables, and outcome measures for study variables were examined using descriptive statistics. A percentage of caregivers' concerns and final diagnosis were correctly identified. Agreement was assessed using kappa coefficient values. The degree of the association between caregivers' concerns and specific developmental disorders was estimated by simple logistic regression. Chi-square tests were used to determine differences in categorical variables, and independent sample *t*-tests were used to examine group differences in other variables.

## Results

### Agreement Between the Caregivers' Initial Concerns and the Professional Identification

Table 2 presents the results of the caregivers' initial concerns and the professional identification. The children's developmental problems identified by caregivers were global (44.7%), followed by speech/language (37.6%), emotional/behavioral (6.1%), motor (6.1%), and cognitive (5.6%) developmental problems, whereas the professions identified global developmental delay (68.0%), emotional/behavioral (9.9%), cognition (4.5%), speech/language (3.9%), and finally motor (2.7%). The mean age at which developmental problems were first noticed was different, with cognition at 55.9 months, emotion/behavior at 45.7 months, global at 43.1 months, speech/language at 31.4 months, and motor at 23.9 months. Overall, the caregivers' initial concerns were the same as the professional diagnosis for 759 children (38.7%), with a discrepancy in 1,204 children (61.3%). Among the 1,204 children, 217 children (11.1%) were finally determined to be typically developing despite the report of various developmental problems by their caregivers. Up to 23.5% of children who initially had motor concerns reported by their caregivers were finally determined to be typically developing, with a statistically significant increase (χ^2^ = 10.84, *p* = 0.013). Of 1,086 children identified at risk for/with a single domain concern, low agreements corresponding to professional identification in each domain were observed ranging from −0.013 to 0.009. The agreement percentages for children identified at risk for/with a multiple domain concern were 72.4% (Kappa coefficient value = 0.010), indicating very poor agreement.

**Table 2:** The caregivers' concerns of children's developmental problems and the results of professional identification (*N* = 1,963).

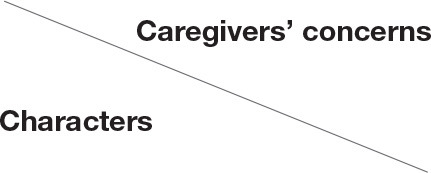	**Single domain concern (*****N*** **=** **1,086)**				**Multiple domain concern** **(***N*** = 877)**	**Total**
	**Cognition**	**Speech/language**	**Emotion/behavior**	**Motor**	**Global/mixed**	
Total number	110 (5.6%)	738 (37.6%)	119 (6.1%)	119 (6.1%)	877 (44.7%)	
Age	55.9 (9.8)	31.4 (10.6)	45.7 (14.6)	23.9 (17.7)	43.1 (14.7)	
Typically developing	13 (11.8%)	101 (13.8%)	12 (10.1%)	28 (23.5%)	63 (7.2%)	217 (11.1%)
**Delay classification**						
Cognition only	11	29	4	5	40	89 (4.5%)
Speech/language only	1	57	0	2	16	76 (3.9%)
Emotion/behavior only	24	27	40	4	99	194 (9.9%)
Motor only	1	11	1	16	24	53 (2.7%)
Global/mixed	60	513	62	64	635	1,334 (68.0%)
*K*-value	0.009	−0.013	0.008	0.007	0.010	
*p*-value	0.004	<0.001	0.015	0.014	0.004	

Table 3 presents the agreement and disagreement of children identified at risk for/with developmental delays corresponding to caregivers' single and multiple concerns. Of 1,086 children identified at risk for/with a single domain concern, delay domain involved in concern domain were emotion/behavior (83.2%), followed by speech/language (68.2%), motor (64.7%), and cognition (60.9%). The major disagreement was observed in the cognition (Kappa coefficient value = −0.010 as indicating no agreement). Among these 110 caregivers who reported that their child had cognitive problem initially, 75 children were finally determined to have emotional/behavioral developmental delay. The major agreement was observed in the speech/language (Kappa coefficient value = 0.418 as moderate agreement). Regardless of caregiver's concerns in speech/language, emotion/behavior, or motor, cognitive developmental delay was the major involved non-concern domain. With respect to 877 children identified at risk for/with a multiple domain concern, the delay domain involved in the concern domain were emotion/behavior (75.0%), followed by cognition (65.6%), motor (64.6%), and speech/language (58.9%). Low agreements corresponding to professional identification in the concern domain were observed. When caregivers reported that their child had cognitive or speech problems initially, emotional/behavioral developmental delay was the major involved non-concern domain. Regardless of caregiver's concerns in emotion/behavior or motor, cognitive developmental delay was the major involved non-concern domain. A possible comorbid presence of cognitive and emotional/behavioral developmental delays should be noticed once caregivers' initial concerns about speech/language development were raised.

**Table 3:** Agreement and disagreement of children identified at risk for/with developmental delays corresponding to caregivers' concerns.

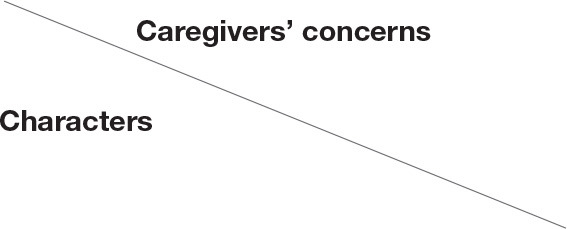	**Single domain concern** ***N*** **= 1,086**			
	**Cognition**	**Speech/language**	**Emotion/behavior**	**Motor**
	***N*** **= 110**	***N*** **= 738**	***N*** **= 119**	***N*** **= 119**
**Delay domain involvement**				
Concern domain	67 (60.9%)	503 (68.2%)	99 (83.2%)	77 (64.7%)
**Non-concern domain**				
Cognitive	–	483	58	64
Speech/language	22	–	24	28
Emotion/behavior	75	396	–	28
Motor	32	231	28	–
*K*-value	−0.010	0.418	0.113	0.181
*p*-value	0.477	<0.001	<0.001	<0.001
	**Global/mixed domain concern** ***N*** **= 877**			
	**Cognition** ***N*** **= 552**	**Speech/language** ***N*** **= 601**	**Emotion/behavior** ***N*** **= 620**	**Motor** ***N*** **= 263**
**Delay domain involvement**				
Concern domain	362 (65.6%)	354 (58.9%)	465 (75.0%)	170 (64.6%)
**Non-concern domain**				
Cognitive	–	137	80	57
Speech/language	29	–	45	27
Emotion/behavior	110	156	–	49
Motor	40	83	56	–
*K*-value	−0.120	0.350	0.200	0.266
*p*-value	<0.001	<0.001	<0.001	<0.001

### The Presence of Specific Developmental Disorders

The presence of cognitive, speech/language, and emotional/behavioral developmental delays was high in this study. Since delays in these developmental domains have been associated with a higher risk of ID, ASD, and ADHD for young children, we further analyzed the presence of ID, ASD, and ADHD among the children in this study. After the evaluation, each child has been diagnosed based on the ICD-10. Based on the ICD-10 coding, F70, F71, and F72 are related to the diagnosis with ID. F84.0 and F84.9 are related to the diagnosis with ASD. The diagnosis with ADHD consists of F90.0 and F90.2. The presence of ID (*N* = 102; 5.2%), ASD (*N* = 412; 21.0%), and ADHD (*N* = 121; 6.2%) was recognized in 1,963 children. Then we analyzed the relationship between caregivers' single and multiple concerns and ID, ASD, or ADHD. For children identified at risk for/with a single domain concern, simple logistic regression revealed that children who were concerned to have speech/language developmental problems were 3.64 times more likely to be diagnosed with ID compared to children without speech/language concerns (Table 4). When caregivers reported their concerns about emotional/behavioral development, their children were 2.77 times more likely to be diagnosed with ASD. When caregivers initially reported their concerns about cognitive and emotional/behavioral developmental problems, their children were 10.36 and 3.94 times more likely to be diagnosed with ADHD, respectively. For children identified at risk for/with a multiple domain concern, children who were concerned to have cognitive developmental problems were 2.01 times more likely to be diagnosed with ID compared to children without cognitive concern. When caregivers reported their concerns about speech/language and emotional/behavioral development, their children were 2.37 and 2.17 times more likely to be diagnosed with ASD, respectively. When caregivers initially reported their concerns about cognitive and emotional/behavioral developmental problems, their children were 2.36 and 2.12 times more likely to be diagnosed with ADHD, respectively.

**Table 4:** Simple logistic regression of caregivers' concerns about developmental domains in children with and without ID, ASD, or ADHD.

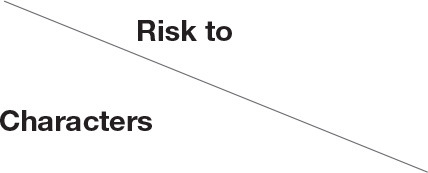	**Single domain concern (*****N*** **= 1,086)**				**Global / Mixed domain concern (*****N*** **= 877)**			
	**Cognition**	**Speech/** **language**	**Emotion/** **behavior**	**Motor**	**Cognition**	**Speech/** **language**	**Emotion/** **behavior**	**Motor**
**ID**								
OR	0.20	3.64	0.38	2.52	2.01	1.65	1.19	1.29
95% CI	0.10–0.40	1.93–6.89	0.18–0.80	0.60–10.58	1.08–3.75	0.87–3.14	0.65–2.20	0.72–2.33
*p*-value	<0.001	<0.001	0.010	0.205	0.029	0.126	0.571	0.391
**ASD**								
OR	0.99	1.00	2.77	0.06	0.67	2.37	2.17	0.38
95% CI	0.60–1.63	0.72–1.38	1.85–4.16	0.02–0.26	0.49–0.93	1.61–3.49	1.47–3.21	0.25–0.56
*p*-value	0.966	0.996	<0.001	<0.001	0.015	<0.001	<0.001	<0.001
**ADHD**								
OR	10.36	0.08	3.94	0.37	2.36	0.38	2.12	0.55
95% CI	5.55–19.33	0.04–0.18	2.03–7.64	0.09–1.54	1.33–4.16	0.23–0.61	1.15–3.93	0.31–0.98
*p*-value	<0.001	<0.001	<0.001	0.170	<0.001	<0.001	0.017	0.044

### Demographic Characteristics Accounted for the Variability

Table 5 presents demographic characteristics related to the agreement and discrepancy between caregivers' single and multiple concerns and professional identification of children's developmental problems. Among those 1,086 children identified at risk for/with a single domain concern, the caregivers' initial concerns were the same as the professional diagnosis for 124 children (11.4%), with a discrepancy in 962 children (88.6%). The age at which developmental problems were first noticed was significantly different, with more children under the age of 3 years in the discrepancy group compared to children in the agreement group (63.8 vs. 53.2%; χ^2^ = 5.27, *p* < 0.05). Compared to children in the agreement group, the number of children in the family was lower, mothers had lower levels of education, and more children belonged to an immigrant family in the discrepancy group. For the other 877 children at risk for/with a multiple domain concern, the caregivers' initial concerns were the same as the professional diagnosis for 635 children (72.4%), with a discrepancy in 242 children (27.6%). The age at which developmental problems were first noticed was significantly different between the agreement and discrepancy groups (41.7 months vs. 46.6 m; *t* = −4.47, *p* < 0.001). More children over the age of 3 years, older mother's age, and higher levels of mother's education were observed in the discrepancy group compared to children in the agreement group.

**Table 5:** The agreement and discrepancy between caregivers' concerns and professional identification of children's developmental problems as a function of demographic characteristics.

**Demographic characteristics**	**Single domain concern**		**Statistics** ***t*** **/χ^2^**	**Global/mixed domain concern**		**Statistics** ***t*** **/χ^2^**
	**Agreement** **(***n*** = 124)**	**Discrepancy** **(***n*** = 962)**		**Agreement** **(***n*** = 635)**	**Discrepancy** **(***n*** = 242)**	
Age (months)	36.7 (14.9)	34.4 (14.9)	1.63	41.7 (14.5)	46.6 (14.5)	−4.47***
5–35 months	53.2%	63.8%	5.27*	35.0%	23.1%	11.31**
36–71 months	46.8%	36.2%		65.0%	76.9%	
**Gender**						
Male	71.8%	71.1%	0.02	76.4%	74.0%	0.55
Female	28.2%	28.9%		23.6%	26.0%	
**Preterm**						
Yes	16.1%	14.2%	0.32	15.6%	17.4%	0.40
No	83.9%	85.8%		84.4%	82.6%	
**First child in the family**						
Yes	56.5%	62.0%	1.40	65.8%	62.8%	0.70
No	43.5%	38.0%		34.2%	37.2%	
Number of children	1.9 (0.7)	1.7 (0.7)	3.07**	1.7 (0.7)	1.8 (0.7)	−1.76
Father's age	37.6 (5.3)	37.2 (5.5)	0.73	37.6 (5.9)	37.8 (5.1)	−0.52
**Father's level of education**						
High school and below	29.0%	36.2%	2.45	41.6%	37.6%	1.15
College and above	71.0%	63.8%		58.4%	62.4%	
Mother's age	35.3 (4.8)	34.5 (4.9)	1.72	34.7 (5.2)	35.5 (4.7)	−2.07*
**Mother's level of education**						
High school and below	21.8%	33.9%	7.35**	39.7%	31.4%	5.13*
College and above	78.2%	66.1%		60.3%	68.6%	
**Marriage**						
Taiwanese couples	98.4%	94.0%	4.10*	93.7%	94.6%	0.27
Immigrant couples	1.6%	6.0%		6.3%	5.4%	

* *p < 0.05*,

** *p < 0.01, and*

*** *p < 0.001*.

## Discussion

The early identification of children younger than 6 years of age at risk of developmental delays is essential for the appropriate early intervention services in Taiwan. This study revealed four main findings. First, the major children's developmental problem identified by caregivers and professionals was global developmental delay. The caregivers' initial concerns were the same as the professional diagnosis for 759 children (38.7%), with a discrepancy in 1,204 children (61.3%). The results showed low agreement between the caregivers and the professionals in all developmental domains. Second, the major disagreement between caregivers' concerns and professional identification was observed in the cognitive developmental domain. Cognitive developmental delay was the major involved non-concern domain across any other caregivers' concerns, indicating a comprehensive evaluation for cognitive development should be conducted in the identification process. Speech/language developmental concern was a common red-flag indicator of cognitive and emotional/behavioral developmental delays. Third, the high rate of presence of ID, ASD, and ADHD was observed. The significant relationships between caregivers' concerns and presence of ID were apparent in speech/language and cognitive development. The presence of ASD was significantly associated with caregivers' concerns about speech/language and emotional/behavioral developmental delay. The presence of ADHD was significantly associated with caregivers' concerns about cognitive and emotional/behavioral developmental delay. Fourth, child's age, the number of children in the family, mothers' level of education, and belonged to an immigrant family were significant indicators for detecting the child's developmental problems among children identified at risk for/with a single domain concern. For caregivers of children identified at risk for/with a multiple domain concern, those with older child, older mother's age, and higher levels of mother's education reported different concerns from professional identification.

The most common developmental delay among the children in our study was a global developmental delay. Consistent with the findings of a previous study, some caregivers usually report their worry about language problems rather than global developmental delay (10). In this study, up to 38.5% of children with global developmental delay were reported as having a speech/language developmental problem by their caregivers initially. Although caregivers appeared to notice their child's subtle developmental signs, they were usually not aware of the different developmental domains comprehensively (10, 12); hence, providing educational guidance about the different developmental domains to parents might be helpful.

In this study, cognitive developmental domain was observed as the major disagreement between caregivers' concerns and professional identification. Notably, we found that cognitive development was the major involved domain associated with global developmental delay. This was due to the fact that the definitive diagnoses for cognitive and global developmental delays overlapped (30). Additionally, cognitive and speech/language developmental delays occurred at a high rate in children with emotion/behavior developmental delay. This is in line with research by Biermann et al. (31) showing that emotion/behavioral developmental delay was associated with cognitive and speech/language developmental problems. However, caregivers usually had difficulties to recognize the signs of potential cognitive or global developmental problems correctly if the child was not severely affected (10, 12). This finding suggests that assessing cognitive development can be considered for every child enrolled in the identification process.

Caregivers' concerns about speech/language developmental delay were significantly associated with the comorbid presence of ID. This is in line with research by Chen et al. (14) showing that caregivers' concerns about speech developmental problems exclusively were noted to yield a high relatively possibility of cognitive developmental problems. Clinical pediatric practitioners should be conscious of recognizing speech/language concerns as a strong indication of identifying cognitive developmental delay.

Recently, the population of individuals with ASD has increased rapidly. According to the Centers for Disease Control and Prevention (CDC) in the United States, this population has increased to twice the size in the previous decade. In our study, the children of caregivers concerned about a developmental delay in various domains had the presence of ASD, with most children with ASD diagnosed at a young age. As expected, language and emotional/behavioral developmental delays were associated with co-occurring ASD (10, 32, 33). Emotional/behavioral developmental concern was the most significant indicator of ASD. Notably, children whose caregivers were concerned about a motor developmental delay were less likely to have ASD in this study. Our findings did not correspond to the findings of Gabis et al. (34), who reported that motor developmental delay is a “red flag” for ASD. One possible reason might be sample characteristics. Among the 119 children at risk for/with motor developmental delay in the present study, nearly a quarter of children were determined to be typically developing, with a small number of children (2.7%) with motor developmental delay and 54% global developmental delay.

The high rate of comorbid presence of ADHD was also observed among children with global developmental delay. As expected, children with cognitive or emotional/behavioral developmental concerns were more likely to have ADHD (35, 36). Caregivers' concerns about cognitive development was the most valid indicator. Previous studies demonstrated that children having deficits in multiple developmental domains can show significant ADHD comorbidity (16, 17). However, children who had caregivers' concerns of speech/language developmental delay were less likely to have ADHD in this study. Previous research indicated that language developmental delays are common in children with ADHD (16, 37), and problems with language comprehension and pragmatics are associated with the core ADHD symptoms such as inattention and impulsiveness (38). Thus, caregivers expressed more concerns about children's cognitive ability rather than their language development.

The findings regarding mother's education levels were in line with previous reports of mother's educational level being critical in the identification of a child's developmental problems (15, 27, 29). Mothers with higher educational levels spent more time teaching or playing with their children than less educated parents (39) and, hence, were more sensitive to detect minor differences because they acknowledged that observing behaviors and discussing their developmental concerns with their pediatricians supported their children's development (29). However, higher levels of mother's education were observed in the discrepancy group compared to children in the agreement group when caregivers reported a multiple domain concern. A possible reason might be sample characteristics. Among the 877 children at risk for/with a multiple domain concern in the present study, 26% of children were determined to be typically developing, and 57.1% were the first child in the family. These mothers might pay much attention on supporting their child's development.

This study has a few limitations. The caregivers and families were recruited from southern cities in Taiwan; hence, geographical differences in socioeconomic status in Taiwan were not considered, and therefore, the findings of this study may not be generalized to families in other areas of Taiwan. Second, the caregivers' concerns about their children's developmental problems and demographic characteristics were collected by caregivers' reports, so caregiver's awareness about their child's development may be overshadowed by their concerns about multiple conditions such as feeding problems, sleeping problems, allergies, subsequent underweight, and activity participation. The caregivers' concerns may be misinterpreted or affected by poor communication.

## Conclusion

The findings of this study provide some indication of the caregivers' concerns about cognitive, speech/language, emotional/behavioral, and motor development that should be questioned in terms of their association with the real problem. We suggest that assessing the cognitive developmental status is essential for all children in the identification process. Speech/language concerns served as a significant red-flag indicator of identifying multiple domains of developmental delay, especially cognitive and emotional/behavioral developmental delays. Furthermore, our findings indicated that practitioners should pay significant attention to educational guidance on increasing the agreement of caregivers' concerns of children's developmental problems with the results of professional identification, especially in the domains of cognitive, speech/language, and emotional/behavioral development. By being aware of caregivers' educational status, the risk of low-level developmental knowledge and utilization of medical support may be reduced. This information may be useful in understanding the discrepancy between caregivers' initial concerns and professional identification in developmental delays.

## Data Availability Statement

The raw data supporting the conclusions of this article will be made available by the authors, without undue reservation.

## Ethics Statement

The studies involving human participants were reviewed and approved by the Institutional Review Board of National Cheng Kung University Hospital. Written informed consent to participate in this study was provided by the participants' legal guardian/next of kin.

## Author Contributions

L-YL and Y-FT conceptualized and designed the study. L-YL performed the statistical analyses, drafted the initial manuscript, and takes responsibility for the paper as a whole. W-HY, W-PL, and C-CC coordinated and supervised the data collection. Y-FT coordinated funding acquisition, methodology, project administration, and supervision. All authors contributed to the article and approved the submitted version.

## Funding

This study was supported by grants from the National Cheng Kung University Hospital (NCKUH-10802015) and the Ministry of Science and Technology (MOST 106-2314-B-006-075 and 108-2314-B-006-066) of Taiwan.

## Conflict of Interest

The authors declare that the research was conducted in the absence of any commercial or financial relationships that could be construed as a potential conflict of interest.

## Publisher's Note

All claims expressed in this article are solely those of the authors and do not necessarily represent those of their affiliated organizations, or those of the publisher, the editors and the reviewers. Any product that may be evaluated in this article, or claim that may be made by its manufacturer, is not guaranteed or endorsed by the publisher.
